# Chemotherapy-Associated Cardiotoxicity: A Silent Threat Evidenced in a Retrospective Cohort Study

**DOI:** 10.7759/cureus.78905

**Published:** 2025-02-12

**Authors:** Felipe A Muñoz-Rossi, Santiago Cárdenas-Corredor, Luis Fernando Saldarriaga Osuna, Diego A Guerra Kunze, Patricia León-León, Donovan A Sánchez, Vanessa Mejia Nates, Luis Felipe Franco Puente, Pamela L Suarez Jaramillo, Antonio J Reche Martinez

**Affiliations:** 1 Internal Medicine, Universidad Nacional de Colombia, Bogotá, COL; 2 Methodology of Health Sciences Research, International University of La Rioja, Bogotá, COL; 3 General Medicine, Universidad Nacional de Colombia, Bogotá, COL; 4 Morphophysiology, Universidad Catolica Santiago de Guayaquil, Guayaquil, ECU; 5 Gynecology, University of Guayaquil, Guayaquil, ECU; 6 Methodology of Health Sciences Research, International University of La Rioja, Quito, ECU; 7 General Medicine, Universidad Católica de Santiago de Guayaquil, Guayaquil, ECU; 8 Emergency, Rey David Clinic, Cali, COL; 9 General Medicine, Universidad del Sinu, Montería, COL; 10 General Medicine, Universidad Libre, Barranquilla, COL; 11 Laboratory Medicine, Puerto Real University Hospital, Cadiz, ESP

**Keywords:** cardio-oncology, cardiotoxicity, chemotherapy, heart failure, left ventricular dysfunction, myocardial injury

## Abstract

Introduction: At present, cancer represents one of the main causes of mortality both globally and in Colombia, with a growing trend that could position it as the leading cause of death shortly, surpassing other diseases of great health impact. Cardiotoxicity associated with antineoplastic treatments can manifest itself at different times, either during the administration of chemotherapy or sometime after its completion, becoming evident only when clinical complications such as heart failure have developed. Therefore, it is essential to use diagnostic tools to identify patients at greater risk of developing cardiotoxicity associated with administering chemotherapeutic agents.

Methods: We conducted a descriptive observational cohort study, which included patients over 18 years of age with an active diagnosis of cancer, both of hematological or nonhematological origin, who were treated in a university hospital in Colombia between 2016 and 2019.

Results: One hundred ninety-seven patients were included, with a mean age of 53. During follow-up, 20 patients (10%) developed cardiotoxicity, with an incidence density of 3.64% person-months. Dyslipidemia was the most prevalent comorbidity (45%), followed by arterial hypertension (28.7%). Non-Hodgkin's lymphoma was the most frequent oncologic diagnosis (40.3%), with an incidence of cardiotoxicity of 13%. Patients exposed to anthracyclines had a higher incidence of cardiotoxicity (11.8%) compared to those not exposed (5.7%), with a relative risk of 2.074 (95% confidence interval = 1.91-2.24). The left ventricular ejection fraction was significantly lower in patients with cardiotoxicity (55.3%) compared to those without cardiotoxicity (62.1%) (p = 0.029).

Conclusions: Taking into account the usefulness of echocardiography and the use of biomarkers found in this study and referred to in the literature, we can determine that these studies, far from being routine, are one of the main strategies that the clinician has to favor the early and timely identification of those patients who are developing a cardiotoxic effect; therefore, it is essential to include these tools in the algorithms of care as a model of serial monitoring. This is not only to reduce the incidence of cardiotoxicity but also as part of an integral management of the oncologic patient to increase the efficiency of pharmacological treatment and improve the quality of life of the patients treated in the short, medium, and long term.

## Introduction

To this day, cancer remains one of the top causes of death around the globe. It is also the second greatest cause of death in Colombia, accounting for about 17% of the overall mortality rate in the nation [1]. It is anticipated that by the year 2030, cancer will overtake cardiovascular disease as the leading cause of death [2].

In this study, we defined cardiovascular toxicity as cancer therapy-related cardiac dysfunction (CTRCD). This broad concept encompasses diverse clinical and subclinical manifestations and reflects the etiologic link to the extensive scope of oncospecific therapies, including chemotherapy, targeted agents, immunotherapies, and radiotherapy [3].

Definitions taken from the European Society of Radiology and Oncology and the International Society of Cardio-Oncology 2022 were taken to primarily define cardiac dysfunction in the setting of oncospecific therapies [3].

Symptomatic CTRCD is characterized by signs and symptoms of heart failure (HF). According to the intensity of the clinical manifestations, it is classified as very severe, severe, moderate, and mild. In the very severe category, HF requires inotropic support, mechanical circulatory support, or consideration of heart transplantation. In the severe classification, hospitalization for HF is needed. For its part, in moderate conditions, outpatient treatment intensification is necessary, especially with diuretics and management of HF. While in mild cases, there are minimal symptoms of HF that do not require intensification of treatment.

On the other hand, asymptomatic CTRCD includes subclinical cardiac dysfunction and is classified into three groups according to left ventricular ejection fraction (LVEF): severe, moderate, and mild. In the severe category, there is an additional reduction in LVEF to values less than 40%. In the moderate group, two scenarios can be presented; in the first, there is an additional reduction in LVEF of ≥10 percentage points, with final values between 40% and 49%; in the second scenario, there is a reduction in LVEF <10 percentage points within the range of 40%-49%, accompanied by a further relative decrease in global longitudinal strain (GLS) >15% from baseline or a further increase in cardiac biomarkers. Finally, in the mild category, LVEF readings are equal to or greater than 50%, with a new relative drop in GLS greater than 15% compared to baseline and/or a new increase in cardiac biomarkers.

It is feasible to clearly describe cardiac dysfunction produced by oncospecific treatment thanks to these criteria, which makes it possible to allow quick diagnosis and appropriate follow-up of patients at risk of cardiotoxicity. Cardiotoxicity is an adverse effect of cancer-specific treatment; consequently, early identification and continuous monitoring of cardiac function in patients undergoing cancer-specific treatment include initial evaluation by detailed clinical history, complete physical examination, and specific diagnostic tests such as echocardiogram, cardiac magnetic resonance imaging, and measurement of biomarkers such as natriuretic peptides and troponin [4,5].

In this scenario, they may include alternative management and monitoring strategies, such as using different cardiac imaging modalities (nuclear imaging and computed tomography) and variations in the frequency and timing of assessments [6]. Interventions can also be compared with traditional management approaches or different cardioprotection regimens.

Drug-induced anticancer cardiotoxicity is influenced by a number of characteristics, such as patient age, previous cardiovascular disease, duration, and type of oncospecific therapy [7]. Therefore, it is essential to have a comprehensive approach in care centers to detect possible cardiovascular complications and treat them promptly to minimize the risk of cardiovascular damage in the medium and long term [8].

The identification of cardiotoxicity consists of a comprehensive assessment of the patient's preexisting cardiovascular risk factors [9]. A limited number of studies have generated different risk scales in selected patient cohorts. However, none of these scales have validation in prospective studies, and in many cases, clinical judgment ends up being a fundamental decision-making tool in certain patient scenarios [10-15]. Therefore, risk assessment should include a complete clinical history with a thorough physical examination and baseline assessment of cardiac function.

Cardiac biomarkers such as natriuretic peptides and troponin should be added to the initial assessment and during follow-up; this approach allows subclinical detection of asymptomatic left ventricular dysfunction and may influence decision-making about the choice of chemotherapy, initiation of cardioprotective measures, or increased periodic patient surveillance [7,16].

Given the above, the initial assessment of the identification of cardiovascular risk factors allows for the proper interpretation of changes during regular monitoring of patients requiring oncospecific treatment.

The different strategies for screening and detecting cardiotoxicity include cardiac imaging (echocardiography, nuclear imaging, and magnetic resonance imaging) and biomarkers [17]. The choice depends on the availability of resources and expertise in their interpretation. The precise timing and frequency of imaging and/or biomarker acquisition will depend on the oncospecific treatment received, cumulative chemotherapy dose, protocol duration, and the patient's baseline cardiovascular status. As part of good clinical practice, certain principles should be followed when ordering cardiac imaging and biomarkers. Some of these principles highlight that tests and modalities with better reproducibility are preferred, images that also provide relevant clinical information are selected, high-quality images without radiation are preferred if available, and finally, the same imaging modality and biomarkers should be used at both initial evaluation and follow-up, as this allows for point-of-care screening and detection [3,18,19].

Oncology treatment, although effective, carries significant risks of cardiovascular toxicity. Since oncology treatment-induced cardiotoxicity can have serious consequences, such as cardiac dysfunction, it is crucial to identify and manage these side effects effectively. Therefore, the objective of the present study is to determine the incidence of cardiotoxicity in adult cancer patients under antineoplastic treatment in a tertiary level hospital in Colombia.

## Materials and methods

A single-center, retrospective cohort study was conducted in a tertiary care institution in Bogotá, Colombia. The data collection period covered three years. The reference population consisted of patients over 18 years of age with an oncologic diagnosis who were treated with chemotherapy on an outpatient or inpatient basis during the study period.

The inclusion criteria include oncologic patients older than 18 years and treated with chemotherapy in a third-level institution in Bogota, Colombia. However, excluded individuals included pregnant patients, those with HIV infection, patients with severe functional and instrumental dependence defined by a Barthel index <50 points and a Lawton scale <5/8, and those whose echocardiographic studies did not comply with Simpson's modified biplane method for ejection fraction or with myocardial deformation measurements by GLS performed with uniform software.

The sample size was calculated using the G*Power 3.1.9.7 program (Heinrich-Heine-Universität Düsseldorf, Düsseldorf, Germany) under an independent mean comparison model with normal distribution. With a confidence level of 95%, a statistical power of 80%, and an effect size of 0.4, 198 subjects were deemed necessary.

Statistical analysis

We began with a univariate analysis to describe the sociodemographic and clinical characteristics of the population. In the bivariate analysis, categorical variables were analyzed using contingency tables and chi-square tests, while continuous variables were described using measures of central tendency, verifying normality with the Kolmogorov-Smirnov test. In the case of normal distribution, Student’s t-tests and Pearson correlation coefficients were used; otherwise, the Mann-Whitney test was used.

As measures of association, relative risks (RR) were calculated, and multivariate analyses were performed with logistic regression models for cardiotoxicity and Cox regression models adjusted for time at risk. Survival curves were generated using the Kaplan-Meier method and evaluated with the log-rank test.

Follow-up included inpatient and outpatient surveillance. Baseline data were collected at patient admission, and follow-up with hepatic profiling was performed until the occurrence of events such as cardiotoxicity, hospital discharge, or death.

Data were analyzed with SPSS version 27.0 (IBM Corp., Armonk, NY) and RStudio (Posit Software, Boston, MA) for visualization, considering a statistical significance level of p < 0.05. Frequency measures included cumulative incidence and incidence rate in the exposed cohort.

Ethical considerations

Considering the retrospective nature of the study, it is a risk-free investigation. No interventions that may modify patient behaviors and treatments will be carried out, so informed consent is not necessary. The confidentiality of the information obtained was guaranteed without mentioning the names of the patients or the medical personnel involved. The health center requested the corresponding authorization to carry out this study. The investigators declare that they have no conflicts of interest.

This research will be carried out in compliance with the fundamental principles established in the Nuremberg Code (1947), the Declaration of Helsinki (1964), and the Belmont Report (1979), which prioritize the well-being and identity of the individuals involved.

## Results

**Figure 1:**
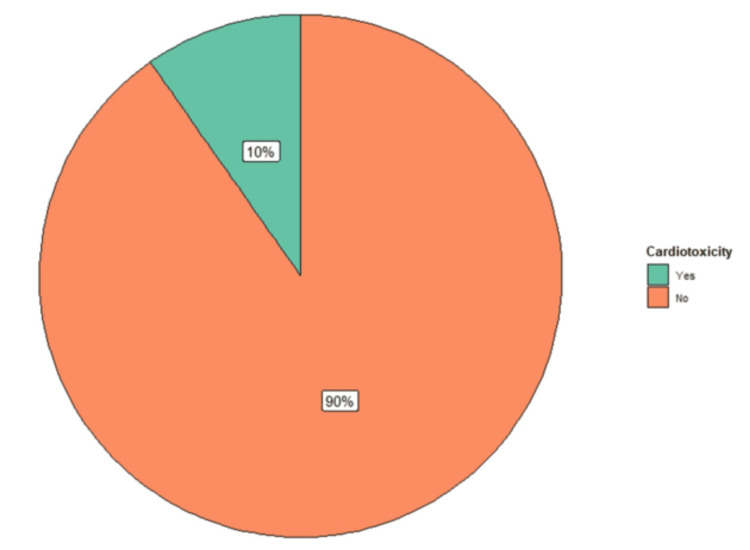
Incidence of cardiotoxicity Data are presented as a percentage Total number of patients: n = 197. Yes: 20 patients (10%). No: 177 patients (90%)

The study included 197 subjects admitted for oncospecific treatment: 109 were men, comprising 55.3% of the population, while 88 were women, representing 44.7%. The mean age was 53 years (95% confidence interval, CI = 50.6-55.4). The sum of the periods at risk was 548.8 person-months. During follow-up, 20 patients (10%) presented with cardiotoxicity (Figure 1), while the incidence density was 3.64% person-months of follow-up.

As for the main comorbidities reported, dyslipidemia was the prevalent one, present in 45% (n = 58) of the subjects, followed by arterial hypertension with 28.7% (n = 37), HF with 14% (n = 27), and peripheral arterial disease with 12% (n = 24) (Table 1).

**Table 1:** Sociodemographic characteristics n is the number of nonmissing values CI: confidence interval

Variables	Values (n = 197)
Age, mean (95% CI)	53 (50.6-55.4)
Sex, n (%)	
Male	109 (55)
Female	88 (47.7)
Comorbidities, n (%)	
Arterial hypertension	
No	92 (71.3)
Yes	37 (28.7)
Dyslipidemia	
No	71 (55)
Yes	58 (45)
Diabetes mellitus	
No	123 (95.3)
Yes	6 (4.7)
Acute coronary syndrome	
No	188 (96)
Yes	7 (3.6)
Atrial fibrillation	
No	185 (95)
Yes	10 (5.1)
Heart failure	
No	168 (86)
Yes	27 (14)
Peripheral artery disease	
No	171 (88)
Yes	24 (12)

According to the oncological diagnoses, the most frequent was non-Hodgkin's lymphoma, with 40.3% (n = 79), with evidence of cardiotoxicity in 10 subjects. The types of cancer are represented in Table 2.

**Table 2:** Types of cancer identified n is the number of nonmissing values

Cancer type	Total, n (%)	Metastasis, n (%)	Ctox, n (%)
Non-Hodgkin lymphoma	79 (40.3)	14 (7.1)	10 (5.1)
Other nonhematological	56 (28.6)	30 (15.3)	4 (2.0)
Other hematological	19 (9.7)	-	2 (1.0)
Breast cancer	17 (8.7)	7 (3.6)	-
Acute myeloid leukemia	11 (5.6)	-	3 (1.5)
Hodgkin lymphoma	7 (3.6)	2 (1.0)	1 (0.5)

**Figure 2:**
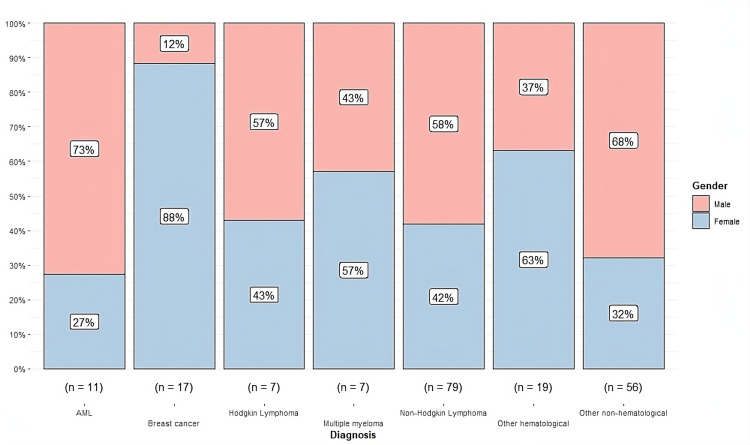
Distribution by gender and type of oncologic diagnosis n represents the number of nonmissing values

A distribution by gender and type of oncologic diagnosis is presented, showing a higher representation in our study population of non-Hodgkin's lymphoma (79/196). Most patients are men, constituting 58% (n = 46), while women represent 42% (n = 33), indicating a notable male predominance in this type of oncologic pathology. On the other hand, as expected, breast cancer was primarily observed in women, accounting for 88% (n = 15), while men represented only 12% (n = 2), consistent with the known epidemiology (Figure 2).

Characteristics of patients with cardiotoxicity secondary to chemotherapy

**Figure 3:**
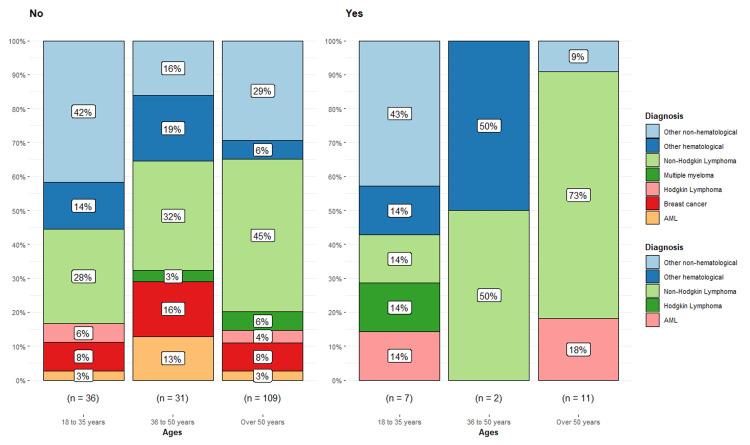
Relationship between ages/cardiotoxicity and oncologic diagnosis Data are presented as a percentage. n is the number of nonmissing values

A total of 55% of the patients in our research who had cardiotoxicity were over 50 years old (n = 11/20), and they were mostly diagnosed with acute myeloid leukemia (AML; 18%, n = 2/11) and non-Hodgkin's lymphoma (73%, n = 8/11) (Figure 3). Notably, neither multiple myeloma nor breast cancer patients had any instances of cardiotoxicity. However, the difference in cardiotoxicity incidence across age groups (16.3%, 6.1%, and 9.1%) was not statistically significant (Pearson's chi-square, p = 0.28) (Table 3).

**Table 3:** Cross table comparing dependent ages and cardiotoxicity A p value of <0.05 is considered significant. The statistical test used was Pearson's chi-square test

Total (n)	18-35 years (n = 43)	36-50 years (n = 33)	Over 50 years (n = 121)	Test statistic	
197	7 (16.3%)	2 (6.1%)	11 (9.1%)	X^2 ^= 2.52; p= 0.28	

Regarding the presence of metastases in oncological patients, no discernible patterns were found that could be related to the appearance of cardiotoxicity. Based on this information, it appears that the existence of metastases does not play a significant role in the development of cardiotoxic adverse effects in this group. Furthermore, no statistically significant differences were identified between the groups, as shown by a Pearson's chi-square value of 1.093 (p = 0.57), indicating no significant correlation between the presence or absence of metastases and cardiotoxicity (Table 4).

**Table 4:** Cross-table comparing dependent metastasis and cardiotoxicity A p value of <0.05 is considered significant

Total (n)	No (n = 142)	Yes (n = 54)	Test statistic
197	15 (10%)	5 (10%)	X^2^ = 1.09; p = 0.57

**Figure 4:**
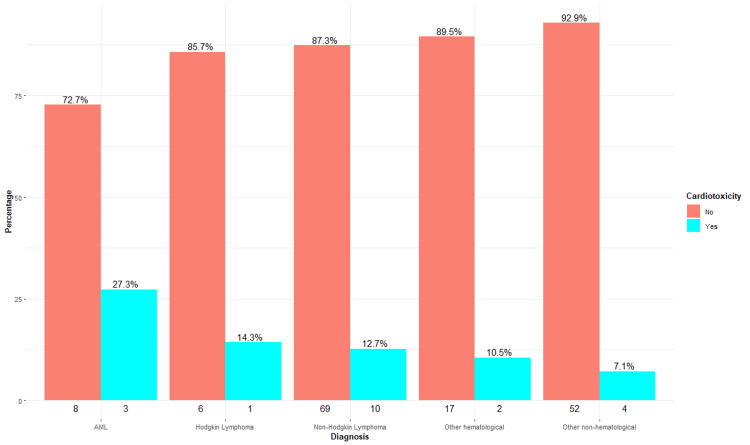
Oncologic diagnoses and cardiotoxicity The numbers below each bar represent the absolute frequency (n) of patients without (left) and with (right) cardiotoxicity for each diagnosis category

In our analysis of cardiotoxicity according to the different oncologic diagnoses, an incidence of 27% (n = 3/11) was observed in patients with AML, while those with Hodgkin's lymphoma and non-Hodgkin's lymphoma showed an incidence of 14% (n = 1/7) and 13% (n = 10/79), respectively. In contrast, patients with multiple myeloma and breast cancer had no cases of cardiotoxicity. On the other hand, miscellaneous hematologic and nonhematologic diagnoses presented a moderate proportion, with 11% (n = 2/19) and 7% (n = 4/56), respectively (Figure 4). These findings highlight the need to monitor these patients under cardio-oncology programs.

**Figure 5:**
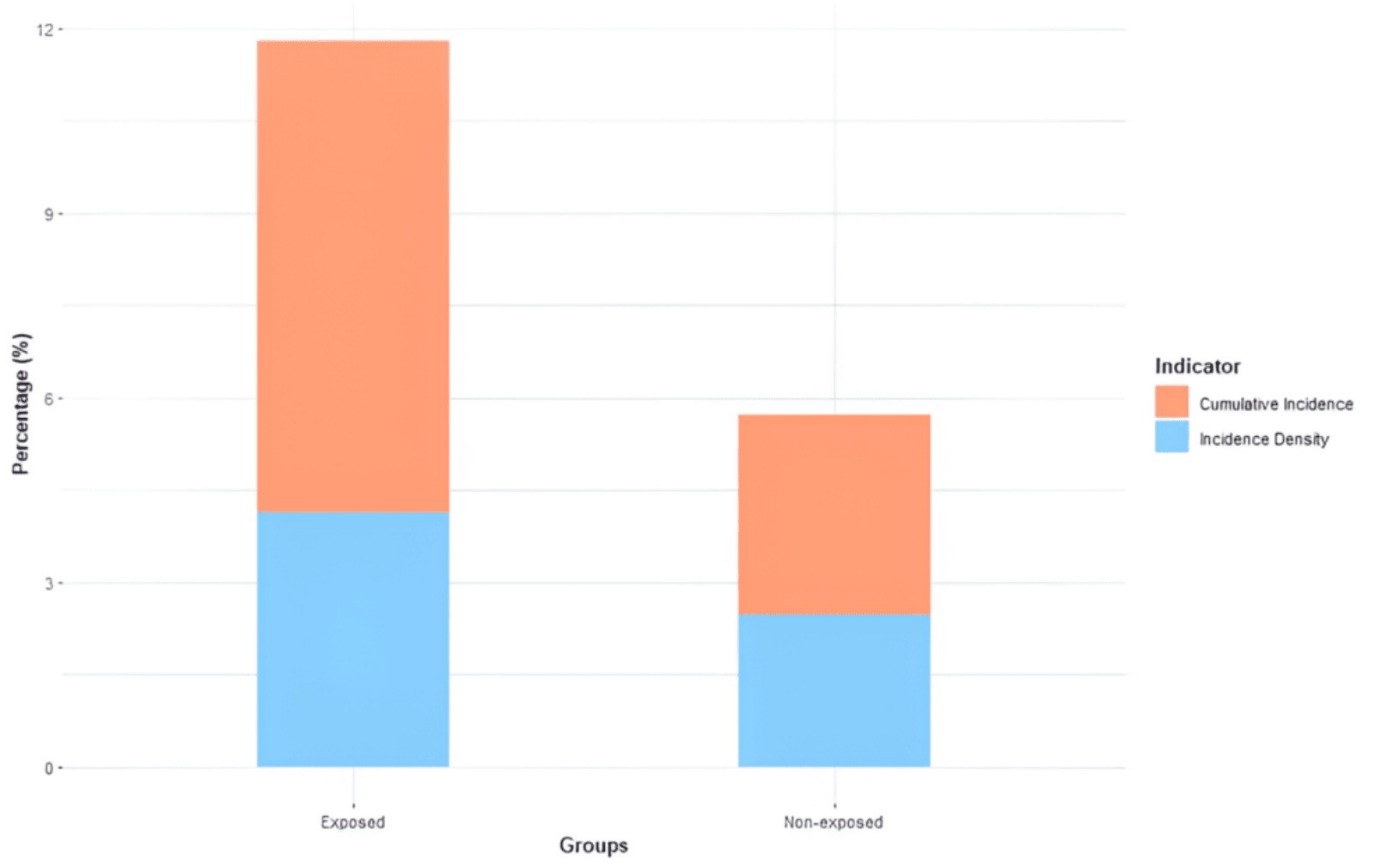
Cumulative incidence and incidence density in anthracycline cardiotoxicity

When the incidence of cardiotoxicity in patients was analyzed according to their exposure to anthracyclines, it was shown that the exposed group had a greater frequency of this problem than the other cohort. Within the group of persons who had not been exposed to anthracyclines, four occurrences of cardiotoxicity were recorded among the 70 individuals. The cumulative incidence of these cases was 5.71%, and the incidence density was 2.47 person-months of follow-up. On the other hand, in the exposed cohort, 16 occurrences of cardiotoxicity were found in 135 patients. This resulted in a considerably higher cumulative incidence of 11.8% and an incidence density of 4.13% person-months of follow-up (Figure 5).

Patients who were given anthracyclines had an RR of cardiotoxicity of 2.074 (95% CI = 1.91-2.24). This indicates that patients who were given anthracyclines had a risk of experiencing this complication that was more than twice as high as patients who were not given this therapy.

**Figure 6:**
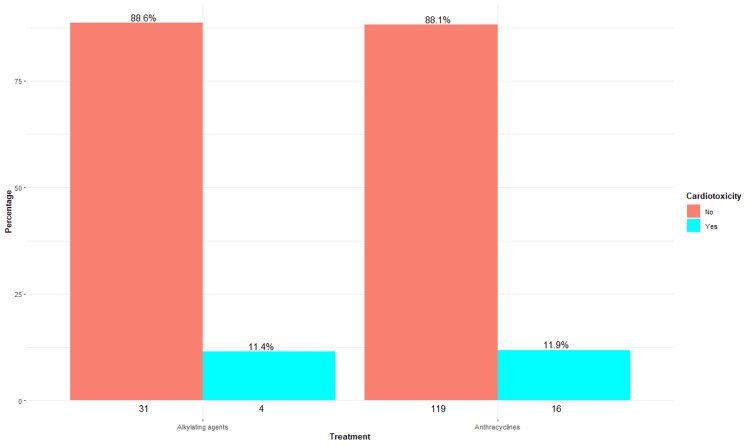
Oncospecific therapy and cardiotoxicity The numbers below each bar represent the absolute frequency (n) of patients without (left) and with (right) cardiotoxicity for each treatment category

Anthracyclines were the most associated therapeutic category with cardiotoxicity, with an incidence of 12% (approximately); however, a similar finding was found concerning alkylating agents, with a cumulative incidence of 11%. In contrast, patients treated with taxanes, antivascular endothelial growth factor (VEGF), antihuman epidermal growth factor receptor 2 (HER2), and antimetabolites showed no cases of cardiotoxicity, which could indicate a possible lower cardiac toxicity related to these agents in this specific cohort (Figure 6).

In patients without cardiotoxicity (solid line), LVEF remains relatively stable as left ventricular mass (LVM) increases, with a tendency to recover at higher LVM values, suggesting that, in the absence of cardiotoxicity, ventricular function can compensate for the increased mass, maintaining an ejection fraction within functional limits.

**Figure 7:**
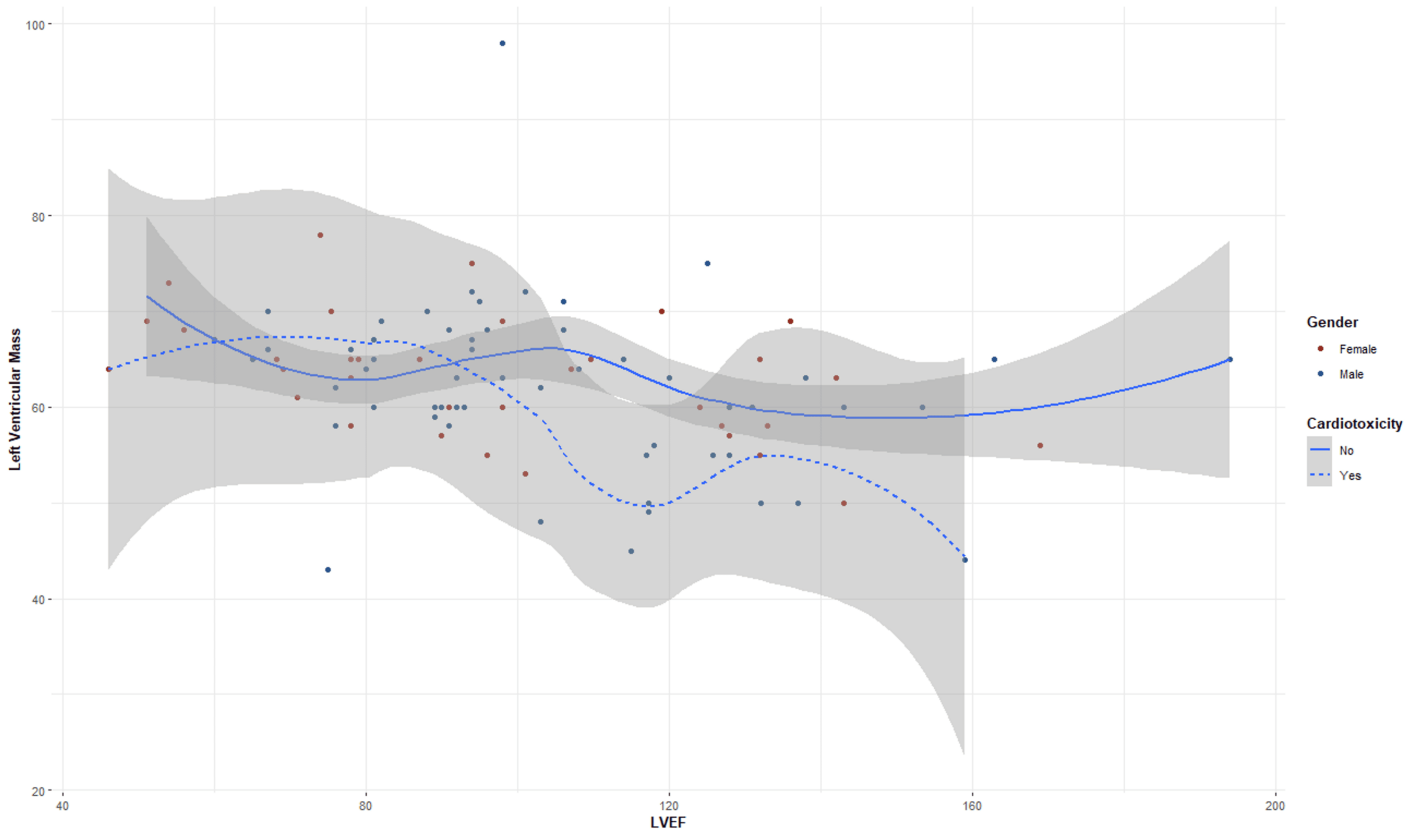
Scatterplot smoothing LVEF/cardiotoxicity LVEF: left ventricular ejection fraction

However, in patients with cardiotoxicity (dotted line), there is a marked decrease in LVEF as LVM increases, probably due to a greater vulnerability of the left ventricle in these patients, secondary to the deleterious effects of the oncospecific treatment on cardiac function, with a statistically significant difference (p = 0.029, Mann-Whitney U test) between both groups with an LVEF of 55.29% (95% CI = 50.0-60.5) in patients with cardiotoxicity and 62.10% (95% CI = 60.79-63.79) in patients without cardiotoxicity, respectively (Table 5). Therefore, the decrease in LVEF in this scenario reveals an increased risk of cardiac dysfunction, reinforcing the need for early interventions and continuous follow-up in patients undergoing oncological treatments (Figure 7).

**Table 5:** Test statistic for LVEF Significant Levene's test (p < 0.05) suggests that variances are not equal LVEF: left ventricular ejection fraction

Test statistic	Statistic	p
Mann-Whitney U test	687	0.029

## Discussion

The present investigation places in evidence the incidence of cardiotoxicity in oncology patients undergoing cancer treatment, with findings similar to those reported by Cardinale et al., which indicated an incidence of 9% in a cohort of 2,625 patients, primarily associated with anthracycline dosage and LVEF at the end of treatment [20]. However, in a meta-analysis of 22,815 cancer subjects, 17.9% developed cardiotoxicity, and 10.9% experienced cardiac events during a 9-year follow-up [21].

In our cohort, the observed incidence is like that reported in previous research, which could suggest a susceptibility of our population to the cardiotoxic effects of oncologic regimens that are not far from studies conducted in developed countries.

In turn, the relationship observed between the presence of metastases and cardiotoxicity was not statistically significant. However, the possible association, together with the impact of oncologic treatment and advanced disease stage, warrants further investigation, as it could contribute to increased cardiac vulnerability.

With respect to ventricular function, patients without cardiotoxicity showed relative stability in LVEF as ventricular mass increased. This phenomenon is explained by the compensatory capacity of the left ventricle in the absence of severe myocardial damage [22,23]. However, in patients with cardiotoxicity, the progressive decrease in LVEF is a function of the increase in ventricular mass, probably due to the deleterious effects of oncospecific treatment. This pattern coincides with that reported by Cardinale et al. [24], who evidenced that changes in LVEF are a sensitive indicator of early cardiac damage in patients treated with chemotherapy.

In accordance with the above, we would like to highlight the need to implement cardio-oncology programs, especially for those patients with oncologic diagnoses that imply an elevated risk of cardiotoxicity depending on their oncospecific therapy. Continuous monitoring and early intervention could decrease cardiac complications, allowing better management of oncologic treatment without compromising patients' quality of life.

Finally, although our findings provide relevant information in our setting, they should be interpreted with caution, given the retrospective nature and sample size in this type of observational study. Future studies with longer long-term follow-ups will be necessary to confirm these observations and explore underlying mechanisms that explain the variability in susceptibility to cardiotoxicity in different types of cancer.

Limitations

Despite the reported findings, we would also like to highlight the following limitations that should be considered when interpreting the results. First, the retrospective nature of the research may lead to selection and reporting biases; therefore, to minimize this, a previous recording of the data was performed, analyzing the quality of the responses to the variable of interest. This allowed the data to be comparable between the groups.

Additionally, a pilot test of the selected variables was carried out in 20 subjects with the collection of information, and according to the results, adjustments were made to ensure the complete collection of information and to evaluate the quality of the information sources.

On the other hand, the sample size of some subgroups, such as those exposed to certain agents like anti-VEGF and anti-HER2, was limited, which prevents robust inferences about cardiotoxicity in these specific groups. In turn, the low incidence of some types of cancer (e.g., AML) and the paucity of patients with metastases may have limited the ability to detect statistically significant differences in these subgroups.

Another element to consider is follow-up time. Although rigorous monitoring was undertaken, the observation time may not have been adequate to identify late cardiotoxic episodes, which might underestimate the cumulative frequency of this problem.

Finally, the absence of a randomized control group inhibits the capacity to demonstrate unambiguous causal links between oncospecific therapy and the development of cardiotoxicity.

## Conclusions

The findings highlight the importance of considering cardiotoxicity as a critical variable in the comprehensive management of oncology patients, especially in those without metastases and exposed to anthracyclines, where closer monitoring could prevent cardiac complications. Furthermore, it highlights the need to implement cardio-oncology departments and cardioprotective strategies that allow exhaustive and individualized monitoring, favoring personalized therapeutic approaches and improving long-term outcomes in this vulnerable population.
